# Vestibulo-perceptual influences upon the vestibulo-spinal reflex

**DOI:** 10.1007/s00221-021-06123-7

**Published:** 2021-05-09

**Authors:** Angela N. Bonsu, Sofia Nousi, Rhannon Lobo, Paul H. Strutton, Qadeer Arshad, Adolfo M. Bronstein

**Affiliations:** 1 Neuro-Otology Unit, Department of Brain Sciences, Charing Cross Hospital Campus, Imperial College London, Fulham Palace Road, London, W6 8RF UK; 2 Division of Surgery, Anaesthetics, and Intensive Care, Department of Musculoskeletal Surgery, Faculty of Medicine, Imperial College London, Charing Cross Hospital Campus, London, W6 8RF UK; 3 inAmind Laboratory, Department of Psychology, Neuroscience and Behaviour, University of Leicester, Leicester, LE1 7RH UK

**Keywords:** Vection, Vestibular-evoked myogenic potentials, Vestibulo-spinal reflexes

## Abstract

The vestibular system facilitates gaze and postural stability via the vestibulo-ocular (VOR) and vestibulo-spinal reflexes, respectively. Cortical and perceptual mechanisms can modulate long-duration VOR responses, but little is known about whether high-order neural phenomena can modulate short-latency vestibulo-spinal responses. Here, we investigate this by assessing click-evoked cervical vestibular myogenic-evoked potentials (VEMPS) during visual roll motion that elicited an illusionary sensation of self-motion (i.e. vection). We observed that during vection, the amplitude of the VEMPs was enhanced when compared to baseline measures. This modulation in VEMP amplitude was positively correlated with the subjective reports of vection strength. That is, those subjects reporting greater subjective vection scores exhibited a greater increase in VEMP amplitude. Control experiments showed that simple arousal (cold-induced discomfort) also increased VEMP amplitude but that, unlike vection, it did not modulate VEMP amplitude linearly. In agreement, small-field visual roll motion that did not induce vection failed to increase VEMP amplitude. Taken together, our results demonstrate that vection can modify the response of vestibulo-collic reflexes. Even short-latency brainstem vestibulo-spinal reflexes are influenced by high-order mechanisms, illustrating the functional importance of perceptual mechanisms in human postural control. As VEMPs are inhibitory responses, we argue that the findings may represent a mechanism whereby high-order CNS mechanisms reduce activity levels in vestibulo-collic reflexes, necessary for instance when voluntary head movements need to be performed.

## Introduction

Vestibular processes contribute to maintaining balance, gaze stabilisation and spatial orientation (Cutfield et al. 2014). These processes are mediated by both low- and higher-level brain mechanisms. Higher-order contributions are thought to be provided by the vestibular cortices, whereas lower-order contributions are affected via brainstem and cerebellum-based circuitry that mediates the vestibular-ocular (VOR) and vestibular spinal reflexes (Cullen 2012; Goldberg and Cullen 2011). Connections between higher- and lower-order process are suggested to implicate the thalamus (Russo et al. 2014).

Recent findings have highlighted that bi-stable percepts, such as binocular rivalry (that contains a motion component) or motion-induced blindness, can modulate long-duration VOR responses involving the velocity storage mechanism (Arshad et al. 2013). However, little is known, whether such high-order neural phenomena can also modulate short-latency vestibulo-spinal responses.

Bi-stable motion perception can be elicited by large-field visual motion in the absence of any physical motion. Two alternate sensations can be perceived, either world motion or self-motion (i.e. vection), two percepts known to be associated with different cortico-subcortical networks (Kleinschmidt et al. 2002). Here, we examine the relationship between the strength of perceived illusionary self-motion (i.e. vection) and the degree of modulation in the amplitude of a short-latency vestibulo-spinal (collic) reflex. A positive finding would indicate that short-latency vestibulo-spinal reflexes can be modulated by higher-order visually induced motion perception mechanisms, likely but not exclusively involving the cerebral cortex (Arshad et al. 2019; Kleinschmidt Andreas et al. 2012; Kleinschmidt et al. 2002; McAssey et al. 2020).

The general relevance of our present research examines whether visual information, which in humans is mostly cortically mediated, can access short-latency brainstem vestibulo-postural mechanisms. Visual motion stimuli can generate powerful postural responses, with and without associated self-motion illusions, but the latency of these responses is in the order of 250–500 ms (Guerraz Bronstein 2008a; b). Some visual modulations of short-latency vestibular spinal responses (ca. 30 ms) have been found by Lacour and colleagues (Lacour et al. 1981). In electromyographic (EMG) potential experiments in monkeys during free fall under different visual conditions, they reported visual modulation of the EMG response “in the interval 60–120 ms but this may occur earlier” (sic) (Vidal et al. 1979). To probe this in man, we can utilise cervical vestibular-evoked myogenic potentials (VEMPs), which are short-latency (< 15 ms) inhibitory EMG responses recorded from contracted neck muscles in response to an acoustic stimulus (Rosengren and Colebatch 2018).

Accordingly, we assessed cervical VEMPs at baseline (no visual motion) and during optokinetic stimulation (see methods section for details). Based on previous findings that bi-stable visual motion percepts can modulate the VOR (Arshad et al. 2013), via cortical inhibition (Arshad 2017), we similarly predict that the recruitment of vestibulo-cortical mechanisms during visually induced self-motion will inhibit vestibulo-collic activity via top–down modulation of the VEMP response.

## Methods

### Participants

Twenty healthy right-handed subjects (9 female) aged between 20 and 45 years (mean age 29.8) with no previous or current history of otological, ophthalmological, neurological or psychiatric disorders (as confirmed by a pre-screening questionnaire) were recruited to participate. The local ethics research committee approved the experimental protocol and all subjects provided written informed consent.

### VEMP recording

The maximum voluntary contraction (MVC) of the sternocleidomastoid (SCM) muscle was assessed whilst the subject was facing forward, for subsequent normalisation purposes. This was performed by instructing the subject to contract their neck with maximum force against a Velcro^®^ strap. This was repeated three times with a 5 s rest interval between each contraction. To elicit VEMP’s, subjects faced 90° to the opposite side being tested and viewed the optokinetic disc used as a visual stimulus (i.e. subjects faced 90° to the right when the left SCM was being tested and vice versa). During VEMP recording, the subject maintained 30% MVC by pushing against the Velcro^®^ strap. Background contraction level was monitored using an EMG biofeedback device (NeuroTrac Simplex unit; Verity Medical Ltd, Braishfield, Hants, UK).

“VEMP” recordings were conducted for both the left and right SCM independently, in a randomised order between subjects. An in-house, custom-built system simultaneously delivered air-conducted sound monaurally into the ear of the stimulated muscle via circumaural headphones. Sound was delivered as 500 Hz clicks at 110 dB nHL (120 dB SPL) of positive polarity, at 200 ms intervals (5/second) with 200 presentations that were averaged to produce a single VEMP response. The acoustic stimulus was given monaurally during three different visual viewing conditions, (i) baseline-static visual stimuli, in this condition the disc did not rotate and remained stationary throughout the trial (ii) clockwise motion and, (iii) anti-clockwise motion. Note, during the “motion” trials, the acoustic VEMP was recorded after 30 s to allow for sufficient time for the development of vection (illusionary sensation of self-motion) in response to the visual stimuli (Brandt, Bartenstein et al. 1998). Furthermore, to prevent any confounding factors, a 30 s laytime was also added to the “non-motion” trial to keep all the conditions uniform.

### Electrode placement

**Fig. 1:**
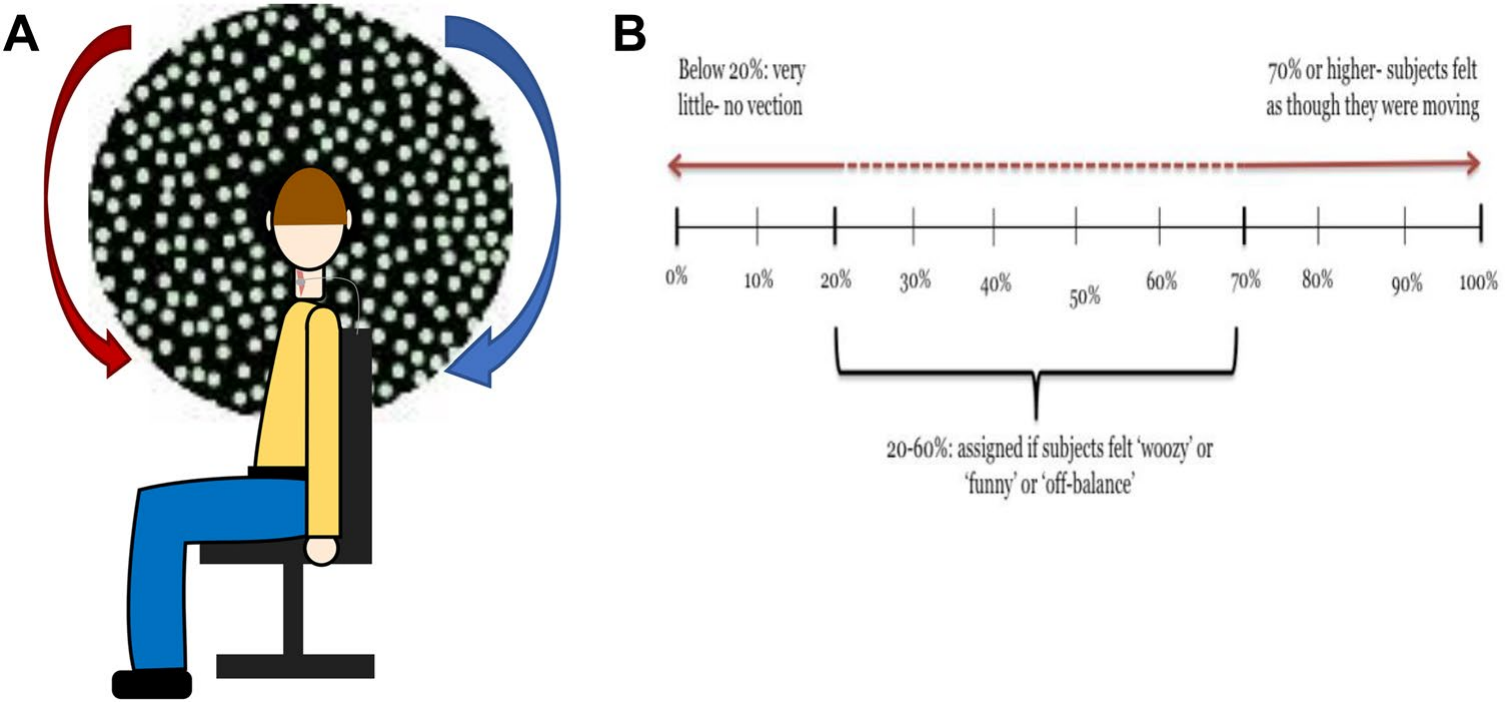
**a** *Schematic Diagram* representing the head positioning of the participant and the electrode montage used to elicit a cervical vestibular-evoked myogenic potential (VEMP). The active surface electrode was placed over the belly of the left and right SCM muscles. Participants were instructed to turn 90° to face the visual stimulus and to maintain tonic contraction of the SCM muscle during the test to elicit an effective VEMP response. Head positioning was maintained using a Velcro^®^ strap. Optokinetic stimulus: participants viewed a pattern of irregularly spaced illuminated white dots on a black background, which moved either clockwise (blue arrow) or anti-clockwise (red arrow). In the static condition the device remained stationary. **b** *Vection scale*. Subjects were asked to assign arbitrary values to the amount of vection that they experienced during the presentation of the visual stimulus

The active surface EMG electrode was placed over the belly of the SCM, the ground electrode was placed over the sternum and the reference electrode was placed over the midpoint of the clavicle. It was ensured that the resistance across all electrodes was less than 5 K ohms. The experiment was conducted in the seating position depicted in Fig. 1a. A Velcro^®^ strap was placed around the subject’s forehead and attached to the chair to stabilise the head (Fig. 1a).

### Optokinetic stimulation

Large-field visual motion stimulation was produced by a rotating disc (diameter of 0.9 m- subtending a visual angle of 84°), which consisted of a pattern of irregularly spaced illuminated white dots on a black background, which moved at a constant angular velocity of 30°/sec in both the clockwise or anti-clockwise direction (Fig. 1a). The disc was controlled via a DC motor with the velocity controlled with a PWM (pulse width modulation) speed regulator. In the static condition the disc remained stationary. The conditions were randomised and repeated twice. Subjects viewed the disc at an eye-target distance of 50 cm from sitting position on the left or right side depending on which SCM was being measured. The centre of the disc was adjusted to eye level. During the experimental procedure, the room was dimly lit, and subjects were always instructed to focus on the center of the disc. In this position with the head turned towards the stimulus, the optokinetic stimulus can generate an illusionary feeling of head and body rotation in the body pitch (sagittal) plane around the visual axis [i.e. falling forwards or backwards (Wolsley et al. 1996)].

### Vection intensity grading

Subjects rated the intensity of vection (illusory self-motion) by assigning arbitrary values (from 0 to 100%) after each condition (Fig. 1b). For guidance, subjects were informed that a score of 70% or over would suggest that they would feel as if their body was moving, tilting or spinning. If the subjects experienced a ‘woozy’, ‘dizzy’, ‘funny’ or ‘off-balance’ feelings (vestibular related sensations) then they were instructed to assign a value, ranging between 20 and 60%. A value below 20% indicated very little or no vection.

### Control experiment

A control experiment was conducted in a separate group of 20 participants to determine whether any visually induced effects on the subjects, particularly vection, could be a non-specific arousal effect. We delivered the same visual stimulation that the subjects viewed through restricted eye shields to reduce the visual field angle by 80%; which critically nullified the ability to perceive vection (Stern et al. 1990). An additional control experiment was issued to add an emotional/arousing component to the control experiment. Here, we induced discomfort with the participants hand submerged in cold water (see below). Thus, the conditions were (i) disc static, (ii) disc rotation with restricted field of view (subjects reported no vection) and (iii) discomfort or pain elicited by submerging the subject’s dominant hand in cold water which varied from 2 to 4° Celsius. The conditions were randomised and repeated twice. Subjects rated the intensity of pain by assigning arbitrary values (from 0 to 100%) after their hand was submerged in cold water. For guidance, subjects were informed that a score of 70% or over would suggest that the cold produced uncomfortable-to-severe pain. A value below 20% indicated no pain and no troublesome coldness.

### Data analysis

The p13 potential was identified as the first distinctive peak in the average waveform, occurring approximately 10–14 ms after stimulus onset, and the n23 potential was identified as the first distinctive trough in the waveform, occurring approximately 19–23 ms after stimulus onset. The peak-to-peak amplitude was derived from the averaged EMG trace for each individual using CED Signal software; (Cambridge Electronic Design, Cambridge, UK).

### Statistical analysis

A 3 × 2 repeated measures ANOVA with factors CONDITION (3 levels—static, clockwise, anti-clockwise) and SIDE (2 levels—left and right SCM) was performed. The aim was to look at how the conditions would independently affect the peak–peak amplitude of the left and right VEMP responses. A subsequent one-way repeated measures ANOVA was carried out on the control experiment to investigate static vs restricted visual motion, vs cold water. The aim here was to look at how the control conditions would affect the VEMP response.

Post hoc *t* tests were performed throughout using Bonferroni corrections. Statistical analysis was performed throughout using IBM^®^ SPSS^®^ Statistics 25.0.

### Correlations

Pearson linear correlations were performed on the % subjective vection rating against the change in VEMP amplitude from baseline (no motion) compared to VEMP amplitude in the (motion trials) main experiment. Subsequent Pearson linear correlations were performed in the control experiment and looked at % subjective pain/level of coldness against the change in VEMP amplitude from baseline (no motion) versus the VEMP amplitude during water submersion.

## Results

### Overview

We observed that the amplitude of the VEMP increased for both the left and right SCM during full-field visual motion, irrespective of direction. Critically, there was no difference in the level of background activity elicited from the SCM muscle, as subjects maintained 30% of MVC throughout. Furthermore, we observed a positive correlation between vection ratings and the change in VEMP amplitude (i.e. comparing measures during motion versus no motion).

### VEMP P1-N1 peak-to-peak amplitude

**Fig. 2:**
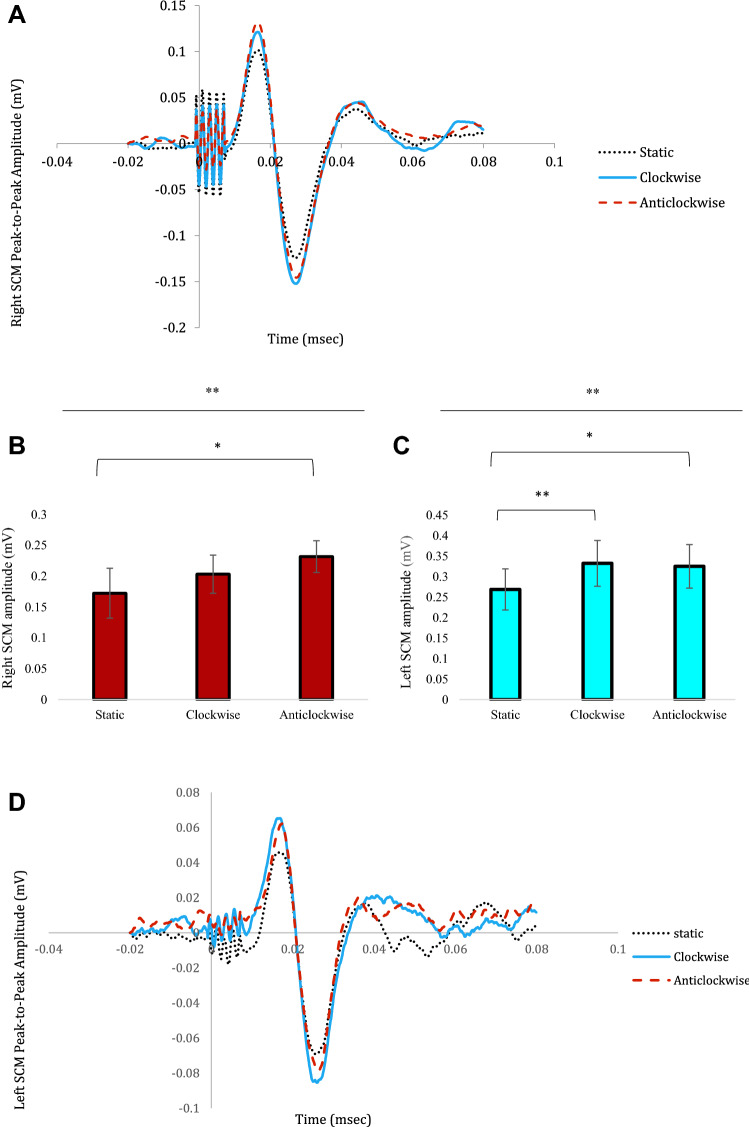
*Changes in VEMP P1-N1 Peak-to-Peak Amplitude. a* Grand average trace for the right SCM for 17 subjects, 3 subject’s traces were removed due to noisy traces. Responses are larger when the disc is rotating clockwise or anti-clockwise as opposed to when the disc is static. **b** Peak-to-Peak amplitude of the vestibular response for the right SCM. **c** Peak-to-Peak amplitude of the vestibular response for the left SCM. **d** Grand average trace for the left SCM for 17 subjects, 3 subject’s traces were removed due to noisy traces; responses are larger when the disc is rotating clockwise or anti-clockwise as opposed to static. Significance levels represented are: * = *p* < 0.05, ** = *p* < 0.01, *** = *p* < 0.001 adjusted for multiple comparisons. Error bars represent ± standard error of the mean (SEM)

A 3 × 2 repeated measures ANOVA revealed a significant main effect of CONDITION (F[2,38] = 7.514; *p* = 0.002). However, there was no significant main effect for SIDE (F[1,19] = 3.139; *p* = 0.092) nor was there a significant interaction between CONDITION*SIDE (F[2,38] = 0.785; *p* = 0.436), (Fig. 2b, c). Post hoc multiple comparisons revealed that there was a significant difference between the static and clockwise conditions (*p* = 0.023) and between the static and anticlockwise conditions (*p* = 0.009), but no significant difference when comparing VEMP amplitudes between the clockwise and anti-clockwise conditions (*p* = 0.999).

**Fig. 3:**
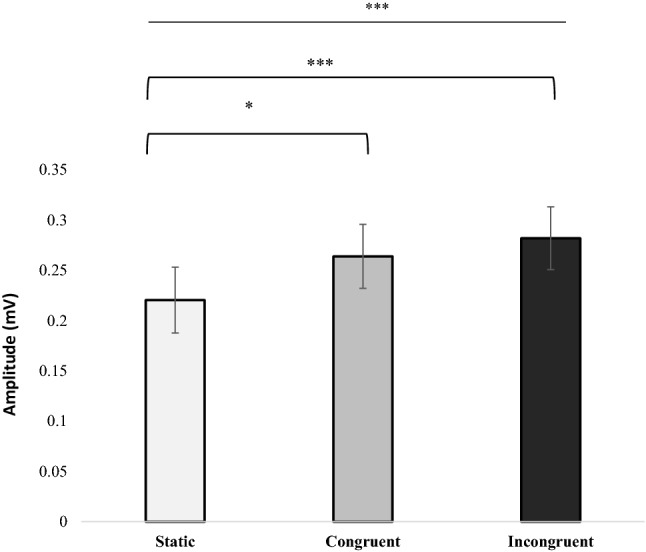
Effect on congruency and incongruency on VEMP P1-N1 Peak-to-Peak Amplitude. Peak-to-Peak amplitude of the vestibular response comparing static to congruency motion and incongruency Significance levels represented are: * = *p* < 0.05, ** = *p* < 0.01, *** = *p* < 0.001 adjusted for multiple comparisons. Error bars represent ± standard error of the mean (SEM)

Given the main effect observed of motion, we conducted an additional repeated measures ANOVA to ascertain if there was a difference in VEMP amplitudes when comparing visual and vestibular activation that were either congruent (i.e. activation of the left SCM due to right head turn and clockwise motion) or incongruent (i.e. activation of the left SCM due to right head turn and anti-clockwise motion). Three measures were taken, (i) static- baseline, (ii) measures during congruency, and (iii) measures during incongruency. Statistical analysis revealed that there was a significant difference between the different conditions (F[2,38] = 8.065; *p* = 0.001), (Fig. 3). Post hoc multiple comparisons revealed that there was a significant difference between static and congruency (*p* = 0.047) and static and incongruency (*p* = 0.001). Critically however, there was no significant difference between congruency and incongruency (*p* = 0.594). The results show an effect of visual motion but lack of a significant directional effect.

### Vection reports and correlation

**Fig. 4:**
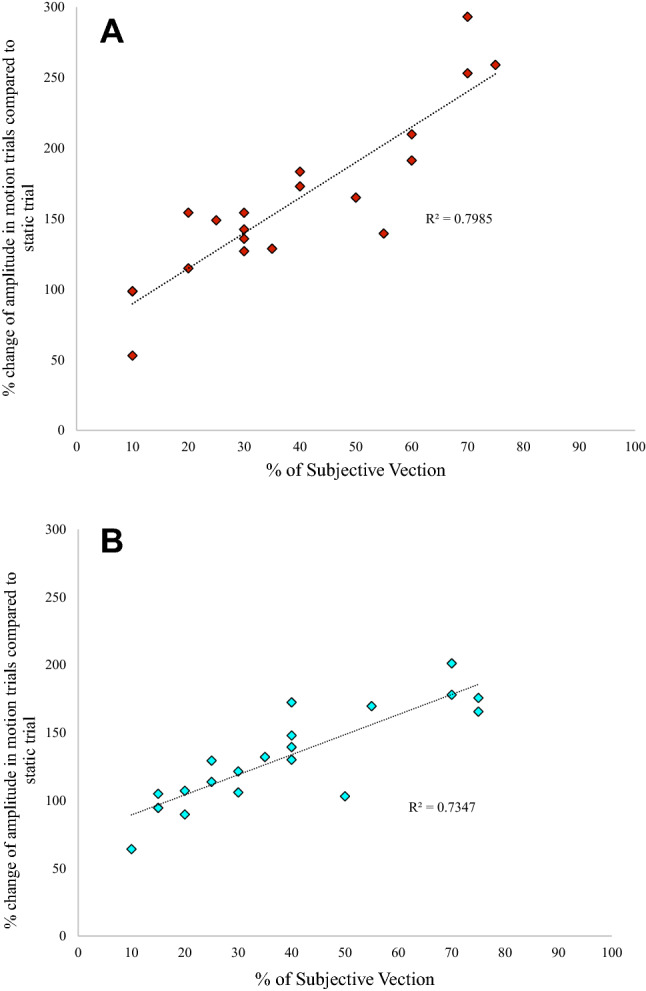
*Vection Correlation.* **a** A significant positive correlation between the amount of vection and the response produced when the amplitude in the motion trials is compared to the amplitude of the static trial in the right SCM. Pearson Correlation 0.894; *p* < 0.0001. **b** A significant positive correlation between the amount of vection and the response produced when the amplitude in the motion trials is compared to the amplitude of the static trial in the left SCM. Pearson Correlation 0.857; *p* < 0.0001

15/20 participants experienced visually induced self-motion during the vection conditions (a score > 20%) with the remaining 5 participants reporting very little to no vection. Figure 4 illustrates the positive correlation observed between the strength of the subjective rating of vection and the normalized peak-to-peak amplitude of the VEMP response (greater vection ratings – > greater peak-to-peak amplitude). Subjects who experienced little or no vection had smaller changes in their VEMP amplitude compared to those that experienced stronger vection sensations. This relationship was observed for both the right (Pearson correlation *r*^2^ = 0.7985; *r* = 0.894; *n* = 20; *p* = 0.0001, Fig. 4a) and left (Pearson correlation *r*^2^ = 0.7347: *r* = 0.857; *n* = 20; *p* = 0.0001, Fig. 4b) SCM.

### VEMP P1-N1 peak-to-peak amplitude on control experiment data

**Fig. 5:**
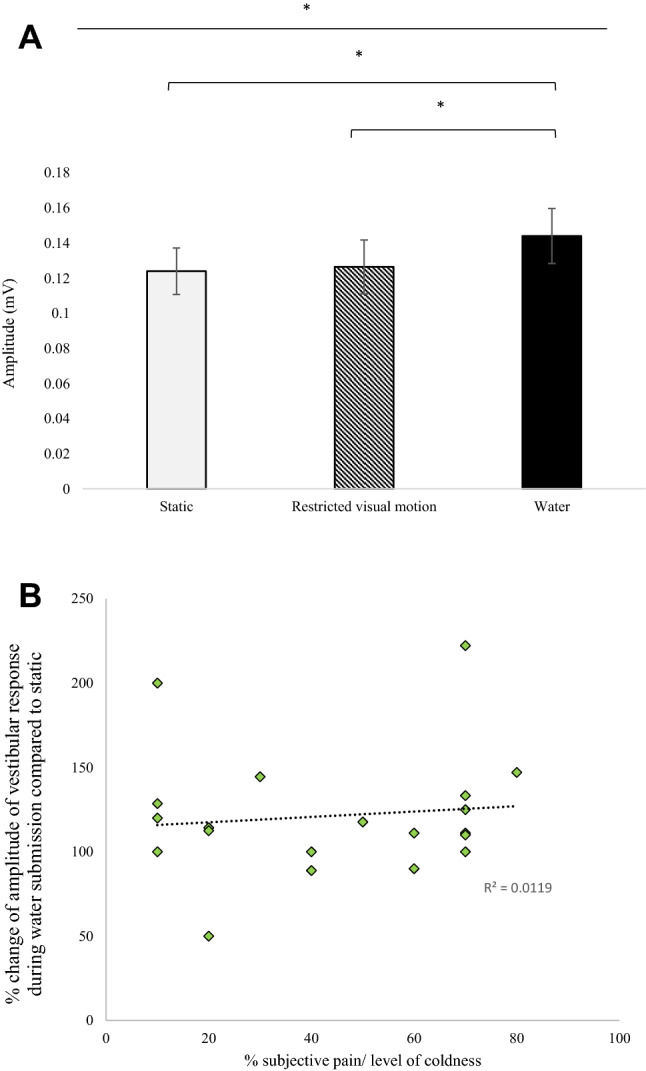
*Control data.* **a** Peak-to-Peak amplitude of the vestibular response for the control data Significance levels represented are: * = *p* < 0.05, ** = *p* < 0.01 adjusted for multiple comparisons. Error bars represent ± standard error of the mean (SEM). **b** Correlation between the level of subjective coldness and the response produced when the amplitude in the water submission trial is compared to the amplitude of the static trial Pearson Correlation 0.109; *p* > 0.05

A one-way repeated measures ANOVA revealed a significant difference for the CONDITION (static, restricted visual motion and cold water) (F [2,38] = 4.914; *p* = 0.013). Post hoc multiple comparisons revealed that there was a significant difference between the static and cold-water conditions (*p* = 0.046) and between the restricted visual motion (no vection) and cold-water conditions (*p* = 0.043). However, there was no significant difference between the static and the restricted visual motion condition (*p* = 0.999), (Fig. 5a).

### Subjective pain report and correlation

10/20 participants rated the intensity of coldness above 50%, whilst the other 10 participants scored the level of coldness as less than 50%. Figure 5b revealed no correlation between the subjective level of cold-induced discomfort and the VEMP amplitude (Pearson correlation *r* = 0.109; *n* = 20; *p* = 0.648). Despite the cold-water stimulus enhancing the VEMP amplitudes, critically, this was not correlative with perceptual measures.

## Discussion

We illustrate that viewing visual motion that engenders illusionary self-motion (vection), modulates the amplitude of the VEMP in a proportional manner to the magnitude of the perceived vection. That is, subjects that experienced greater illusory self-motion exhibited a larger increase in VEMP amplitude from baseline.

Our findings agree with our initial hypothesis that bi-stable motion perception would activate vestibular cortical networks and in-turn modulate vestibulo-collic reflexes. According to our previous work on vestibulo-ocular mechanisms (Arshad et al. 2013), this modulation seems to be via top-down inhibitory mechanisms, and this is interesting because VEMPs are an inhibitory response that reduces the amount of pre-existing contraction in sternomastoid muscles (Colebatch and Rothwell 2004) (Rosengren et al. 2010). Thus, the increased VEMP amplitudes found imply enhanced visual motion-mediated suppression of vestibulo-collic activity. It must be borne in mind that vestibulo-collic (and cervico-collic) reflexes must be suppressed during head movements initiated at higher levels in the neuraxis, including voluntary head movements (Goldberg and Cullen 2011). Short- and middle-latency vestibulo-collic and vestibulo-reticular inhibitory pathways (Wilson and Schor 1999) may be involved in this effect. Hence, the significance of our findings is having identified a mechanism whereby vision and visually mediated self-motion can inhibit vestibulo-collic activity levels.

VEMPs originate in the vestibular system, likely the sacculus (Rosengren et al. 2010). As such VEMPs would be expected to be modulated by real movement or tilt which modify excitability levels of the vestibular receptors and pathways. However, the specific phenomenon we report highlights that even visually induced illusory self-motion perception is capable of modulating vestibulo-collic reflex mechanisms (Guerraz Michel and Bronstein 2008a, b). The presence of such perceptually correlated effect illustrates how visual and vestibular control of posture are under high-order CNS control. Such control facilitates the integration of multiple sensory cues to maintain spatial orientation and postural control in a dynamic environment. This notion is supported by a multitude of previous findings but, more relevant to the current results, by experiments showing that vection illusions increase the magnitude and directional accuracy of visually evoked postural responses (Thurrell and Bronstein 2002). Although visual modulation of postural and vestibular function was generally known, the current research adds that such visual modulation is not only exerted upon long-latency, long-duration systems, e.g. vestibular velocity storage-related (Arshad et al. 2013) or perceptual Merfeld and Zupan 2002), but also on brief and short-latency vestibulo-spinal mechanisms. Our results show that vestibulo-spinal reflex responses latencies < 15 ms can be modulated by visual motion stimuli, which are shorter than the modulation of vestibulo-spinal reflexes previously reported in baboons during freefall at 30 ms latency (Lacour et al. 1981; Vidal et al. 1979). It remains to be seen whether transient visual stimuli (as opposed to continuous, as used here), are also able to modify VEMP amplitudes, and at what latency.

Although the conscious perception of visually mediated self-motion (i.e. its qualification as dizziness or spinning and its rating) can only be mediated by the cerebral cortex, animal work has established that large-field rotating visual stimuli as required to elicit circular vection activate vestibular neurons in the brainstem, thalamus and cerebellum as well cortical areas (Brandt et al. 1998a, b). It is this cortical-subcortical network of visuo-vestibular neurons that is thought to underlie the emergence of conscious vection. Thus, it would be theoretically plausible that the visual effects on VEMPs take place subcortically, only in parallel to the strength of the vection illusion. As we have no way of disentangling this, we have taken the pragmatic approach of ascribing VEMP modulation to the actual variable measured, subjective vection intensity.

It is worth noting the absence of any directional effects in our data, that is, both clockwise and anti-clockwise visual stimulations modulated right and left VEMPs similarly. We have postulated above that the visual motion-mediated modulation of VEMPs likely represents a high-order mechanism reducing neck muscle activity levels via increasing activity in short-latency inhibitory vestibulo-collic pathways. The findings therefore indicate that this neck EMG suppressive mechanism is non-directional, perhaps simply paving the way for oncoming voluntary head movements or complex postural responses. Both of these movements are initiated by higher levels mechanisms and will require downregulation of vestibulo-collic activity levels. Appropriately, at the point of vection onset, oculomotor (Thilo et al. 2002) and postural studies (Thurrell and Bronstein 2002) describe changes in motor strategy, and fMRI studies report selective activation of cortical and subcortical brain structures (Kleinschmidt et al. 2002). An alternative explanation for the absence of directional effects in our data would be provided by the hypothesis that a reasonable response of the head control system when facing motion stimuli would be to simply modulate background levels of muscle tone (Gresty 1987). However, if visual motion and vection simply recruited such a mechanism, we would have seen enhanced background EMG levels during visual motion, but we did not as background EMG levels remained constant.

It could be argued that vection modulated the VEMP response via other non-specific mechanisms, such as attention or arousal. Supporting such a proposition is previous work illustrating that VEMP amplitudes are increased by threat-induced anxiety, fear, and arousal (Naranjo et al. 2016). To control for this in our current study, we performed a supplemental experiment that elicited the VEMP response during, (i) viewing the same visual stimuli with a restricted visual field to supress vection and (ii) submersion of the hand in cold water. Our results revealed that arousal by submersion of the hand in water but not central viewing visual motion modulated the VEMP response compared to baseline measures. Thus, we can directly rule out the fact that visual motion per se mediated the response but not arousal.

Accordingly, a component of the modulation we report could be attributed to an arousing effect, whereby central modulation of the vestibular nucleus complex occurs through excitatory inputs from neural centres involved in emotional processing (Naranjo et al. 2016). Although the arousal component is likely to have a small effect on the increase in the VEMP response, as shown here and in previous work (Naranjo et al. 2016), we propose that the major contributing factor to the modulation we report herewith, is mediated by vestibulo-perceptual influences signalling head rotation and directly impacting upon postural control mechanisms. Supporting this notion is the highly significant visual effect found here as well as the strong and direct correlation between modulations of VEMP amplitude with vection ratings. The cold-induced effect was, in contrast, smaller, only borderline significant and showed no correlation with subjective ratings of coldness or discomfort.

To conclude, we demonstrate that VEMPs responses can be proportionally modulated by the strength of perceived visually induced self-motion. It is not visual motion per se but the strength of the vection illusion that mediates this effect. Thus, our findings indicate that visual stimuli can influence fast acting vestibular reflexes like VEMPs.

## Data Availability

Requests should be made to the corresponding author.

## References

[CR1] Andreas K, Philipp S, Geraint R (2012). Variability of perceptual multistability: from brain state to individual trait. Philos Trans Biol Sci.

[CR2] Arshad Q (2017). Dynamic interhemispheric competition and vestibulo-cortical control in humans; a theoretical proposition. Neuroscience.

[CR3] Arshad Q, Nigmatullina Y, Bronstein AM (2013) Handedness-related cortical modulation of the vestibular-ocular reflex. J Neurosci Off J Soc Neurosci 33(7): 3221. https://www.ncbi.nlm.nih.gov/pubmed/23407975 10.1523/JNEUROSCI.2054-12.2013 PMC6619206 23407975

[CR4] Arshad Q, Ortega MC, Goga U, Lobo R, Siddiqui S, Mediratta S, Bednarczuk NF, Kaski D, Bronstein AM (2019). Interhemispheric control of sensory cue integration and self-motion perception. Neuroscience.

[CR5] Brandt T, Bartenstain P, Janek A, Dieterich M (1998). Reciprocal inhibitory visual-vestibular interaction. Visual motion stimulation deactivates the parieto-insular vestibular cortex. Brain (london, England: 1878).

[CR6] Brandt T, Bartenstein P, Janek A, Dieterich M (1998). Reciprocal inhibitory visual-vestibular interaction. Visual motion stimulation deactivates the parieto-insular vestibular cortex. Brain J Neurol.

[CR7] Colebatch JG, Rothwell JC (2004). Motor unit excitability changes mediating vestibulocollic reflexes in the sternocleidomastoid muscle. Clin Neurophysiol.

[CR8] Cullen KE (2012). The vestibular system: multimodal integration and encoding of self-motion for motor control. Trends Neurosci.

[CR9] Cutfield NJ, Scott G, Waldman AD, Sharp DJ, Bronstein AM (2014). Visual and proprioceptive interaction in patients with bilateral vestibular loss. NeuroImage Clin.

[CR10] Goldberg J, Cullen K (2011). Vestibular control of the head: possible functions of the vestibulocollic reflex. Exp Brain Res.

[CR11] Gresty M (1987). Stability of the head: studies in normal subjects and in patients with labyrinthine disease, head tremor, and dystonia. Mov Disord.

[CR12] Guerraz M, Bronstein AM (2008). Ocular versus extraocular control of posture and equilibrium. Neurophysiol Clin Clin Neurophysiol.

[CR13] Guerraz M, Bronstein AM (2008). Mechanisms underlying visually induced body sway. Neurosci Lett.

[CR14] Kleinschmidt A, Thilo KV, Büchel C, Gresty MA, Bronstein AM, Frackowiak RSJ (2002). Neural correlates of visual-motion perception as object- or self-motion. Neuroimage.

[CR15] Lacour M, Vidal PP, Xerri C (1981). Visual influences on vestibulospinal reflexes during vertical linear motion in normal and hemilabyrinthectomized monkeys. Exp Brain Res.

[CR16] McAssey M, Dowsett J, Kirsch V, Brandt T, Dieterich M (2020). Different EEG brain activity in right and left handers during visually induced self-motion perception. J Neurol.

[CR17] Merfeld DM, Zupan LH (2002). Neural processing of gravitoinertial cues in humans. III. Modeling tilt and translation responses. J Neurophysiol.

[CR18] Naranjo EN, Cleworth TW, Allum JHJ, Inglis JT, Lea J, Westerberg BD, Carpenter MG (2016). Vestibulo-spinal and vestibulo-ocular reflexes are modulated when standing with increased postural threat. J Neurophysiol.

[CR19] Rosengren SM, Colebatch JG (2018). The contributions of vestibular evoked myogenic potentials and acoustic vestibular stimulation to our understanding of the vestibular system. Front Neurol.

[CR20] Rosengren SM, Welgampola MS, Colebatch JG (2010). Vestibular evoked myogenic potentials: past, present and future. Clin Neurophysiol.

[CR21] Russo A, Marcelli V, Esposito F, Corvino V, Marcuccio L, Giannone A, Conforti R, Marciano E, Tedeschi G, Tessitore A (2014). Abnormal thalamic function in patients with vestibular migraine. Neurology.

[CR22] Stern RM, Hu S, Anderson RB, Leibowitz HW, Koch KL (1990) The effects of fixation and restricted visual field on vectioninduced motion sickness. Aviat Space Environ Med 61(8):712. https://www.ncbi.nlm.nih.gov/pubmed/2400374 2400374

[CR23] Thilo K, Guerraz M, Bronstein AM, Gresty MA (2002). Percept-related changes in horizontal optokinetic nystagmus at different body orientations in space. Exp Brain Res.

[CR24] Thurrell A, Bronstein A (2002). Vection increases the magnitude and accuracy of visually evoked postural responses. Exp Brain Res.

[CR25] Vidal PP, Lacour M, Berthoz A (1979). Contribution of vision to muscle responses in monkey during free-fall: visual stabilization decreases vestibular-dependent responses. Exp Brain Res.

[CR26] Wilson VJ, Schor RH (1999). The neural substrate of the vestibulocollic reflex. Exp Brain Res.

[CR27] Wolsley CJ, Sakellari V, Bronstein AM (1996). Reorientation of visually evoked postural responses by different eye-in-orbit and head-on-trunk angular positions. Exp Brain Res.

